# Drawing or gesturing? Prior knowledge moderates the impact of embodied pedagogies

**DOI:** 10.1186/s41235-026-00740-y

**Published:** 2026-07-13

**Authors:** Icy Yunyi Zhang, Zoey Zhao, Rui Yu, Jennifer Tan, Ji Y. Son

**Affiliations:** 1 UW Madison Educational Psychology, University of Wisconsin, Madison, 53706 USA; 2 University of California, Los Angeles, USA; 3 California State University, Los Angeles, USA

**Keywords:** Embodied cognition, STEM education, Prior knowledge, Gesture, Drawing

## Abstract

Educators face persistent challenges in fostering deep understanding of abstract STEM concepts. Theories of embodied cognition propose that grounding abstract ideas in bodily action and perception can enhance learning. Teachers often intuitively use embodied pedagogies such as gesturing and hand-drawing in the classroom, both by performing these themselves or asking students to do these actions, yet the comparative effects of these different types of embodied pedagogies are not well understood. Furthermore, the effect of these pedagogies needs to be put into perspective of students’ varying levels of prior understanding. It is unlikely that a single pedagogy will suit all learners. Building on research examining how embodied pedagogies interact with prior knowledge, we contrasted gesture-based and hand-drawing-based instruction in a college-level introductory statistics and data science course. Students were randomly assigned to view instructional videos that enacted either drawing or gesture then reenacted the demonstrated actions. Results showed that prior knowledge significantly moderated instructional effectiveness: For low-prior-knowledge learners, gesture-based instruction outperformed drawing-based instruction, whereas no difference emerged for high-prior-knowledge learners. The findings of this study contribute to our theoretical understanding of how embodied pedagogies impact learning and offer practical insights for tailoring embodied pedagogies to learners’ prior knowledge in the classroom.

## Significance

Understanding how students learn abstract concepts in STEM is both a theoretical and practical challenge. Theories of embodied cognition argue that grounding abstract ideas in bodily action can support learning, but not all forms of embodiment are equivalent. This paper advances the field by systematically comparing two embodied pedagogies common in the classroom—gesturing and hand-drawing. Importantly, the study highlights that the effectiveness of these pedagogies depends on learners’ prior knowledge. Using a data-driven approach, we established an empirical threshold to distinguish low- from high-prior-knowledge learners. We then tested the impact of gesture- versus drawing-based instruction in the laboratory with students who were taking a college-level introductory statistics and data science course. Results showed a clear interaction: For learners with low prior knowledge, gesture-based instruction significantly outperformed drawing, suggesting that sensorimotor engagement provides crucial support when foundational knowledge is limited. In contrast, for high-prior-knowledge learners, both pedagogies were equally effective, indicating that once a sufficient knowledge base is established, the difference in sensorimotor engagement may be less important or different affordances may serve as interchangeable supports. Theoretically, these findings refine our understanding of the mechanisms of embodied learning by showing that performing pedagogical movements do not equally benefit low-prior-knowledge learners. Practically, they offer clear guidance for tailoring embodied pedagogies to learners’ needs: Instructors may want to prioritize gesture-based instruction for novices while leaving drawing-based approaches for more advanced learners. Together, this work demonstrates the value of mechanism-aware, learner-centered embodied pedagogies for STEM education.

## Introduction

Discovering pedagogies that help learners develop robust, deep, and coherent understanding of abstract concepts in Science, Technology, Engineering, and Mathematics (STEM) domains is a persistent goal in education (National Academies of Sciences, Engineering, and Medicine, 2018). Concepts in STEM domains, such as mathematical models and formulas, are considered abstract because they typically capture invariant structural information among diverse and, importantly, superficially dissimilar situations. For example, the same basic regression equation can describe relationships in very different contexts, such as predicting a person’s height from foot size or predicting a country’s life expectancy from its gross domestic product. Scholars have referred to such concepts as *idealized* because they are stripped of real-world complexities and expressed in generalized forms (Koedinger et al., 2008). While idealization makes concepts broadly applicable, it can also render them more difficult for learners to grasp, as the connection to concrete experience is less apparent.

Theories of embodied cognition suggest one way to address this difficulty: by grounding abstract ideas in action and perception (Nathan, 2021). These theories argue that the body is not just a vessel for the mind but a key driver of higher-order thinking, reasoning, and problem-solving, emphasizing that cognition is grounded in the interplay between body and mind (Hutto & Abrahamson, 2022; Lakoff & Johnson, 2008; Lindgren et al., 2022). These perspectives—whether described as embodied, enactive, ecological, embedded, or extended cognition—converge on the idea that perception and action shape conceptual understanding (Barsalou, 1999; Clark, 2008).

Building on this foundation, embodied pedagogies, broadly defined as instructional methods that involve bodily actions and representations in the learning process (Duijzer et al., 2019), can improve learning by grounding abstract concepts in perceptual and motor systems (Borghi & Pecher, 2011; DeSutter & Stieff, 2017; Golonka & Wilson, 2012; Johnson-Glenberg et al., 2016; Zhang et al., 2022). Rather than relying solely on abstract, amodal representations such as symbols or equations detached from sensory experience (Nathan, 2012) embodied pedagogies can help students develop more meaningful, personally relevant, and robust understandings by connecting abstract STEM concepts to concrete bodily experiences (Barsalou, 2008; Nathan & Alibali, 2021; Shapiro & Spaulding, 2014; Shapiro & Stolz, 2019; Zhang et al., 2022).

A growing body of research highlights the potential benefits of embodied pedagogies. Studies have shown that engaging in bodily actions such as gesturing (e.g., Broaders et al., 2007; Walkington et al., 2014), directed actions (e.g., Goldin-Meadow et al., 2009; Nathan et al., 2021; Zhang et al., 2021), hand-drawing (e.g., Beach et al., 2023; Fiorella & Mayer, 2016; Zhang et al., 2025a), and object manipulations (e.g., Congdon et al., 2018; Fuhrman et al., 2021; Reed, 2018) can enhance learning compared to abstract instruction alone. Related work further suggests that observing or even predicting actions also supports learning when compared to non-embodied pedagogies (e.g., Bokosmaty et al., 2017; De Koning & Tabbers, 2011; Valenzeno et al., 2003; Xia et al., 2025; Zhang et al., 2022). However, engaging the body is not uniformly effective. Learning improves when bodily activity is conceptually aligned with the target content, whereas mismatched actions can hinder understanding (Gunter et al., 2015; Zhang et al., 2021). Thus, it is not movement per se that matters, but how action meaningfully maps onto conceptual structure.

Beyond conceptual alignment, the specific forms of actions learners perform might also influence learning outcomes, especially when considering the differences in learner characteristics (Zhang et al., 2025b, 2026). Increasing evidence suggests that embodied pedagogies can have heterogeneous effects depending on learner characteristics such as prior knowledge and working-memory capacity (Congdon et al., 2018; Congdon, 2024; Congdon & Goldin-Meadow, 2021; Fyfe et al., 2015; Zacharia et al., 2012; Zhang et al., 2024a, 2025b, 2026). Toward this end, understanding how different embodied pedagogies support learning, and for whom, is therefore an important next step for both theory and instructional design.

This question is practically urgent. In classrooms, teachers routinely gesture and draw as part of everyday explanation. In addition to using gestures or drawing themselves, teachers often prompt students to actively reproduce these actions—for example, asking students to show with their hands how the slope increases, or to draw a residual line on their worksheet before proceeding. However, these practices are typically implemented without explicit consideration of the distinct affordances they may provide for learners with different levels of prior knowledge. Without evidence-based guidance, decisions about when to gesture, when to sketch, or when to use other embodied supports may be driven by habit or convenience rather than by an understanding of how these activities shape learning.

In the present study, we focus on two widely used, low-cost embodied pedagogies: gesture and hand-drawing. Although both involve bodily movement, they differ in aspects such as representational function. Gestures are transient movements in which action itself constitutes the representation, often highlighting motion or relational change over time. Drawing, in contrast, uses movement to produce a persistent external artifact that can be inspected and revised; here, motor activity serves primarily to create a stable visuospatial representation. Although drawing may require finer motor control, this motor activity is typically instrumental to creating a stable visual artifact rather than constituting the representation itself.

Statistics and data science provide an especially relevant context for this comparison. Core ideas in these domains are frequently communicated through spatial and dynamic representations, and instructors commonly gesture and draw over data visualizations to illustrate relationships. Comparing these two pedagogies in this context can therefore advance theory while offering directly actionable guidance for STEM instruction.

Below, we will review prior work on each pedagogy and on comparative studies where available, and explain why learners’ prior knowledge might moderate the effectiveness of these two pedagogies.

### The effectiveness of gesture in STEM

Past research has demonstrated the effectiveness of gesture and hand-drawing individually in STEM domains. Research has examined both learner-produced gesture and instructor-produced gesture in STEM domains, with converging evidence that gesture can support conceptual learning, particularly when it is aligned with the underlying concept (e.g., Alibali & Nathan, 2012; Alibali et al., 2014; Cherdieu et al., 2017; Stieff et al., 2016; Wakefield & Goldin-Meadow, 2021; Zhang et al., 2021). Importantly, the benefits of learner-produced gestures extend beyond spontaneous co-speech gestures to enacted gestures performed after instruction. For example, Cherdieu et al. (2017) found that students who reproduced an instructor’s gestures after watching an anatomy lesson showed better recall of structure names and spatial locations on delayed tests than students who only watched but did not reproduce the gestures.

Although fewer studies have examined gestures specifically within statistics and data science, existing evidence similarly suggests that both instructor-generated and learner-produced gestures can support learning. For example, Rueckert et al. (2017) found that undergraduate students who viewed a statistics lesson on the conceptual foundations of ANOVA accompanied by instructor gesture demonstrated greater learning gains than students who received identical verbal instruction without gesture. Similarly, Zhang et al. (2021) examined the effects of performing directed actions while watching a statistical lesson on mean and variation. Learners who performed content-matched actions demonstrated greater conceptual understanding than those who performed mismatched movements or did not move.

### The effectiveness of drawing in STEM

STEM learners who draw during learning demonstrate improved understanding of visual–spatial content (Ainsworth et al., 2011; Fan, 2015; Quillin & Thomas, 2015). A review conducted by Fiorella and Zhang (2018) suggests that learner-generated drawing (e.g., giving learners an opportunity to draw during, between, and after instruction) is generally more effective than passive strategies such as reading alone or other text-focused activities for improving comprehension and transfer. A review by Wu and Rau (2019) suggests that student-generated drawing activities improve STEM learning because they externalize thinking and engage learners in a sequence of cognitive and sociocultural processes that deepen conceptual understanding. Although research has mainly focused on drawing as a generative activity (Fiorella & Mayer, 2015; Leutner & Schmeck, 2014), other research shows that watching an instructor draw through an instructional video can also improve learning (e.g., Fiorella & Mayer, 2016; Fiorella et al., 2019; Quillin & Thomas, 2015).

Specifically, in the domain of statistics and data science, students who viewed instructional videos in which statistical representations were dynamically drawn demonstrated greater conceptual understanding of the normal distribution than students who viewed static diagrams (Zhang et al., 2025a).

### Comparing different types of embodied pedagogies and the moderating role of prior knowledge

How do these two embodied pedagogies compare against each other? So et al. (2014) directly compared the effects of gesture and drawing on spatial learning by asking participants to rehearse routes using either hand gestures in the air or by drawing the routes on paper. The results showed that both gesture and drawing improved recall relative to only mental rehearsal without bodily actions or no rehearsal, indicating that both forms of bodily activity can support encoding of spatial information. However, participants who gestured demonstrated more accurate route recall than those who drew.

Beyond the specific comparison of gesture versus hand-drawing, other research has compared the effects of various embodied pedagogies (e.g., Bokosmaty et al., 2017; Calvo-Merino et al., 2006; Cook & Goldin-Meadow, 2006; Goldin-Meadow et al., 2012; James & Swain, 2011; Kronke et al., 2013; Schwartz & Plass, 2014; Sommerville et al., 2005; Zhang et al., 2025b). For example, one study by Beach and colleagues (2023) compared the effect of hand-drawing, object manipulation, and reviewing a 3D anatomy application (control) on first-year medical students’ learning of spinal anatomy. Participants in the drawing group used paper and pencil to draw and label the assigned anatomy, while those in the object manipulation group used clay, string, and Styrofoam disks to model and label it. Both groups used a 3D app as a reference. The researchers found that only participants in the hand-drawing group improved significantly from pretest to posttest. Interestingly, object manipulation—despite offering higher sensorimotor engagement—did not lead to significant learning gains. However, because the total sample size was only 62, the researchers also pointed out the potential lack of power for this study.

A crucial insight from this set of literature is that learners’ prior knowledge of the concepts may moderate the effectiveness of the embodied pedagogies (Zhang et al., 2025b, 2026). Instructional approaches that provide more concrete grounding or action-based support may particularly benefit learners with lower prior knowledge. Notably, much of this evidence comes from research with children (e.g., Beach et al., 2023; Congdon & Goldin-Meadow, 2021; Congdon et al., 2018; Zacharia et al., 2012). For example, in a study by Congdon and Goldin-Meadow (2021), children with low prior knowledge who received abstract gesture instruction performed worse on a posttest than both (1) their high-prior-knowledge peers who received the same abstract instruction and (2) low-prior-knowledge peers who learned from concrete gesture instruction.

Far less research has examined whether these mechanisms generalize to postsecondary learners, who possess greater symbolic fluency and are expected to learn abstract concepts in more complex subject domains. Zhang et al. (2026) addressed this question in a longitudinal study in a college-level introductory statistics course. College students were randomly assigned to perform or observe embodied activities (e.g., manipulating paper datasets, gesturing on visualizations of statistical models, drawing deviations and sums of squares). Prior knowledge significantly moderated instructional effectiveness: Low-prior-knowledge learners benefited more from observing, whereas high-prior-knowledge learners benefited more from performing.

Together, these findings suggest that both developmental stage and prior knowledge may shape how embodied pedagogies function. But more work is needed to put into perspective the heterogeneous effects of different embodied pedagogies for learners with varying levels of prior knowledge, especially in the context of higher education. Clarifying these patterns will refine theoretical accounts of embodiment while providing more actionable guidance for integrating these approaches into everyday instructional practice.

### Rationale for the current study

In the current project, we compared the effectiveness of gesture and hand-drawing for learners with low versus high prior knowledge in the context of statistics and data science education. This domain is ideally suited for such a comparison: It is highly visual, involving visual representations of data and statistical models. Both gesture and drawing are natural pedagogical interventions for working with such representations. Introductory statistics is also one of the most widely taken college courses in the USA (enrolling about 1.5 million students annually according to Digital Promise), and the skills targeted—interpreting models and visualizations—have broad real-world relevance.

Our instructional content for this 1-hour study was the empty model, which does not consider group membership and uses the grand mean as the prediction, and the two-group model, which takes group membership into account and makes separate predictions for each group (the group means). Students were also taught how to calculate the sum of squares error for each model as a measure of model fit. These concepts align with long-standing calls to center modeling in statistics instruction (e.g., Rodgers, 2010; Joe & Fountain, 1996), a shift reflected in recent curriculum innovations (e.g., CourseKata.org; Judd et al., 2017; Kaplan, 2017; Son et al., 2021). Importantly, the participants of the study were college students who were enrolled in an introductory statistics and data science course. Thus, the content of the intervention built on concepts they were already learning in the course.

We developed nine short instructional video clips in each condition to teach these modeling concepts. In each clip, the instructor either drew or gestured upon a data visualization while explaining the concept. Participants watched each video clip, repeated the sentence spoken by the instructor by reading out the text printed on the screen, and then reenacted the instructor’s actions.

Although in this watch-read-enact sequence, it might seem unnatural to read and then enact, consider the task from a novice’s perspective: Reproducing the movements while simultaneously trying to read the sentence is extremely difficult. This would likely push cognitive load past reasonable limits and even result in false reproduction of the movements. Having participants reenact an action immediately after reading the sentence is a form of deliberate rehearsal that reasonably preserves the temporal proximity and conceptual alignment between speech and action while managing cognitive load. Although this introduces a brief temporal separation between speech and learner-produced action, research has shown that such a sequence can enhance retention and conceptual integration (e.g., Cherdieu et al., 2017; Yang et al., 2014).

In addition, although gesture and drawing can differ along many dimensions in naturalistic contexts, the narration, instructional content, and timing of gesture and drawing were tightly controlled in the current study. After these controls, gesture and drawing in the context of the current study differed in two main ways. First, they differed in the functional role of movement: In gesture, movement itself constituted the representation, whereas in drawing, movement was instrumental in producing an external, symbolic artifact. For example, gesture participants produced a horizontal line with their hand while drawing participants drew a horizontal line. Consequently, in drawing, the hand posture was constrained by tool use (holding a pen), and representational meaning was conveyed primarily through the marks produced on the graph. Second, drawing generated a persistent visuospatial trace available for inspection, whereas gesture was transient and disappeared once enacted. It is important to note that the study does not test the isolated effect of one specific mechanism such as embodied representation or representational permanence, but rather compares two embodied instructional formats that differ in how action externalizes relational structure.

An important issue for the current investigation concerns how to distinguish learners based on prior knowledge. Simulation-based theories of embodied learning propose that multimodal frames created through learners’ previous physical experiences might be particularly important (Barsalou, 2008). The existence of previous mental representations (or not) suggests a threshold perspective: Once learners reach a certain level of prior knowledge, extensive sensorimotor engagement may no longer be necessary for them to benefit from instruction. Below and above this threshold, variability in prior knowledge may play a smaller role. Consequently, many studies in embodied learning research classify learners into low- versus high-prior-knowledge groups (e.g., Congdon et al., 2018; Fyfe et al., 2015; Zacharia et al., 2012). However, because these studies only involved the learning of one single, relatively easy concept (e.g., scale of measurement or balance scales), they separated learners based on whether they had a correct versus incorrect understanding of that concept. In contrast, our study targets more complex content, making the division between low and high prior knowledge less straightforward. Therefore, we recruited a separate sample of novice students who had not yet encountered modeling-related concepts in the domain to establish a threshold for differentiating low and high prior knowledge.

We hypothesized that gesture instruction would be more beneficial than hand-drawing instruction for low-prior-knowledge learners, as having movement itself constitutes the representation may support dynamic grounding of unfamiliar concepts. This hypothesis is supported by theories of embodied learning, which suggest that mapping abstract concepts onto temporally structured movement can support learning, particularly for learners with limited prior knowledge (Sommerville et al., 2005; Zhang et al., 2021). In addition, in the previously reviewed study that directly compared gesture and hand-drawing, So et al. (2014) propose that gesture may facilitate learning by directing attention to relational movement sequences during rehearsal, whereas drawing may shift attention toward producing a visual product. At the same time, drawing may also offer advantages due to its representational permanence. By generating a stable visuospatial trace that remains available for inspection, drawing may ease cognitive load. Thus, although we predicted an advantage for gesture, the possibility remains that persistent externalization may play a critical role in early learning.

Predictions for high-prior-knowledge learners are less certain, given the limited research on this group. However, we conjecture that when learners already have sufficient background knowledge, drawing may better support learning by providing stable visual anchors and facilitating deeper conceptual connections.

## Method

### Participants

A total of 188 college students taking an introductory statistics class taught using a modeling framework (see more at coursekata.org) at two large public institutions in the same metropolitan area in the United States participated in the main study: one highly selective, research-intensive institution and one access-oriented public university. They were randomly assigned to one of two conditions: gesture condition in which they reenacted gestures demonstrated in the instructional video clips (n = 92); drawing condition in which they reenacted drawings (n = 96). Out of 188 participants, 150 identified as female (79.8%), 37 as male (19.7%), and 1 as third gender or non-binary (0.5%). Participants were ethnically diverse: 57 Asian or Pacific Islander (30.3%), 8 Black or African American (4.3%), 54 Hispanic (28.7%), 21 of multiple ethnicities or other (11.2%), 2 who preferred not to say (1.1%), and 46 White or Caucasian (24.5%).

### Materials

Two sets of instructional materials (videos accompanied by identical paper worksheets) were created: one for the Gesture condition and one for the Draw condition. Both sets are aimed to teach the statistics concepts of the general linear model (GLM), the empty model, the two-group model (a model with a categorical predictor variable), and sum of squares.

In each set of instructional materials, there were nine videos, identical in narration and differing only in the actions visually depicted. The videos ranged in length from 22 seconds to 1 minute 27 seconds, with a total duration of 7 minutes 54 seconds. A detailed summary of each video’s content and length is provided in Appendix A. Video clips were recorded with an overhead camera to show the instructor’s hand gesturing or drawing on top of the worksheet. In each clip, the instructor narrated and performed the assigned action (gesturing or drawing) sequentially rather than simultaneously. After each spoken sentence, the instructor executed the corresponding action and then returned to narration. The separation was intended to direct participants’ attention to the narration, which was critical for understanding the underlying conceptual ideas.

**Fig. 1:**
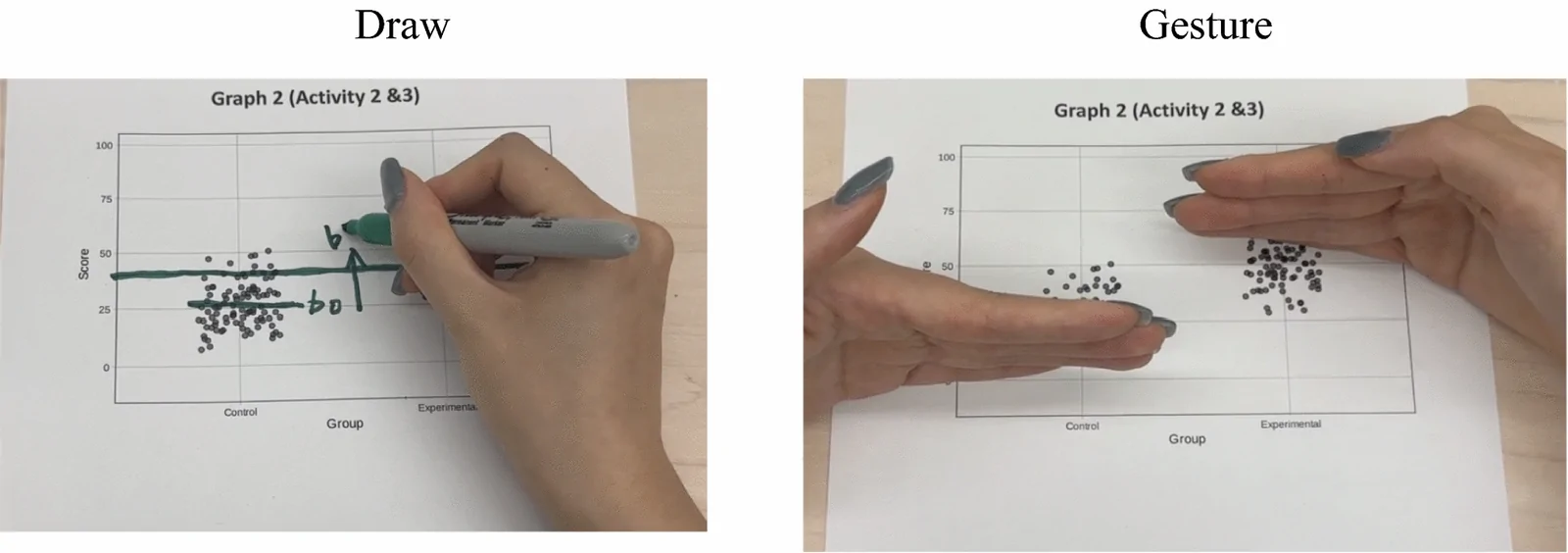
Example screenshots of the videos in each condition

For example, in one section of the clip the narration said, “The b1 is around 25, which is represented as the difference between the model predictions of the two groups.” In the Draw condition, the instructor drew a line between the model predictions of the two groups. In the Gesture condition, the instructor moved their hand(s) up and down in the distance between the model predictions of each group. Screenshots of videos in each condition are shown in Fig. 1. Two example videos can be accessed through the project’s OSF page: https://osf.io/zth3g/?view_only=4539759962164c8085b3acda1d854ad4

### Design and Procedures

Upon entering the laboratory, participants were seated at a laptop with printed worksheets beside it. After receiving an explanation of the procedures, they read and signed the consent form. They then completed a set of pretest questions (see a complete list of the pretest questions in Appendix B) and proceeded to watch a series of instructional videos. After each video, which included narration and a hands-on demonstration (either gesture or drawing), participants advanced to a screen displaying the narrated sentence as text. They read the sentence aloud and reenacted the demonstrated action using the worksheet. This process was repeated for each video. After completing the series, participants took a posttest consisting of multiple-choice and short-answer questions assessing the concepts taught (see a complete list of the posttest questions in Appendix C). Lastly, participants completed a postsurvey that asked them to reflect on their experience and rate how engaged they were during the instructional activity on a 1 to 7 Likert scale (i.e., while reenacting the actions from the instructional videos).

A trained experimenter oversaw the session, assisting participants with troubleshooting and clarifying any confusion. The experimenter’s responsibilities included welcoming participants, explaining the setup, monitoring the session, providing corrections when necessary, and ensuring that the experiment was completed in full. After the pretest, the experimenter closely monitored the participant through the first video and reenactment sequence to catch any potential errors. Once participants completed this first sequence successfully, the experimenter stepped back, continuing to observe more passively while remaining available to address questions or clarify instructions.

In rare cases when a participant failed to vocalize the narrated sentence, the experimenter reminded them to read the sentence aloud before reenacting. Similarly, if the participant read the text and performed the action simultaneously, the experimenter asked the participant to repeat the sentence and the action separately.

### Measures

#### Pretest and prior knowledge group

The pretest consisted of ten true–false items designed to evaluate students’ understanding of the concepts targeted in the instructional materials, including the general linear model, the empty model, the two-group model, and sum of squares. Of the correct answers, seven were “true” and three were “false.” A complete list of the pretest questions and the number of participants who answered each question correctly are provided in Appendix B.

To address the absence of a standardized threshold for classifying prior knowledge on this measure, we established an empirical benchmark using a separate novice sample. This approach allowed us to avoid a purely sample-dependent median or mean split in the main experimental sample and to anchor the classification in what students could achieve with only minimal exposure to the target concepts. Specifically, we administered the same pretest to 62 college students (47 female, 15 male) from a public university who had no prior training in a modeling-based approach to statistics. These participants first received brief, definition-based descriptions of the target concepts and then completed this pretest using Qualtrics. The novice sample’s average score was 6.65 out of 10 (*SD* = 1.64), so we used 7 as the cutoff: Participants in the main experiment who scored below 7 were classified as having lower prior knowledge, whereas those who scored 7 or higher were classified as having higher prior knowledge. This cutoff differs from a chance-performance benchmark. For example, although a binomial test indicates that 8 correct responses would be required to reject the null hypothesis of random guessing at approximately *p* <.05, that benchmark addresses whether performance exceeds random responding. In contrast, our goal was to identify a boundary between performance consistent with guessing plus surface-level familiarity and performance suggesting relatively stronger initial understanding.

#### Posttest

The posttest consisted of 22 questions (15 multiple-choice questions, seven open-response questions). A complete list of the posttest questions is shown in Appendix C. The open-response questions were graded by two trained coders blind to the participants’ conditions: 0 for incorrect, 1 for correct, and 0.5 for correct but incomplete or exhibited minor misunderstandings.

After each coder independently graded all student responses, we calculated their percentage of agreement. A discrepancy was defined as any disagreement on a single response (e.g., one coder assigning a score of 0.5 and the other a 1). If discrepancies exceeded 10% for any question (i.e., agreement fell below 90%), a re-grading procedure was triggered.

Regrading began with a revision of the scoring rubric. To inform this process, two coders compiled all discrepant responses and jointly reviewed them to identify ambiguities or inconsistencies in the rubric that may have contributed to the disagreements. The rubric was then revised to ensure consensus on the previously discrepant cases and clear agreement on the updated criteria. Using the revised rubric, both coders independently re-graded all responses for that question, followed by a second round of agreement checks. This cycle continued automatically until inter-coder agreement reached at least 90% for each question.

For instance, re-grading was triggered for posttest Questions 6b and 6 d, which asked students to compare different measures of variability across two data sets: the sum of squares reduced by the model, which we will refer to as SSM (6b), and the total sum of squares from the null or empty model, which we will refer to as SST (6d). Because one dataset had less variability overall, the correct answer for 6 d (SS Total) was “overall data variance is smaller,” and this was awarded full credit. The original rubric also gave partial credit for the same statement on 6b (SSM), even that item also required reference to the variance explained by the model. Coders observed that many students had exactly the same response to 6b and 6d. This suggested a lack of conceptual understanding of SSM, which led coders to give different scores (0 versus 0.5). To address this, the rubric was revised in a face-to-face meeting so that responses referencing only overall variance on 6b received no credit. This revision increased inter-coder agreement to over the agreement threshold. After all re-grading was complete, the two raters reached agreement on 99.92% of responses. Remaining discrepancies were resolved through joint discussion.

## Results

Based on our empirical threshold, the participants in the experiment were separated into the low-prior-knowledge group (n = 43) and the high-prior-knowledge group (n = 145). Because participants in the experiment were all learning the modeling concepts in a course, making their prior understanding of these concepts different from that of complete novices, there were more high-prior-knowledge students than low-prior-knowledge students.

As a check of random assignment, a logistic regression was conducted to examine whether prior knowledge groups (low vs. high) differed by condition. Participants’ prior knowledge group did not differ significantly by condition, B = 0.37, *z* = 1.05, *p* =.292, odds ratio = 1.45.

Before evaluating the moderating role of the prior knowledge group, we conducted an analysis of covariance to examine the effect of condition on posttest performance while controlling for prior knowledge. The condition did not significantly predict posttest scores, *F*(1, 185) < 0.01, *p* =.990, proportional reduction in error (PRE) <.001. In contrast, prior knowledge was a significant predictor, *F*(1, 185) = 10.00, *p* =.002, PRE =.051, with high-prior-knowledge participants scoring higher on the posttest than low-prior-knowledge participants.

### The moderating role of learners’ prior knowledge on posttest performance

**Fig. 2:**
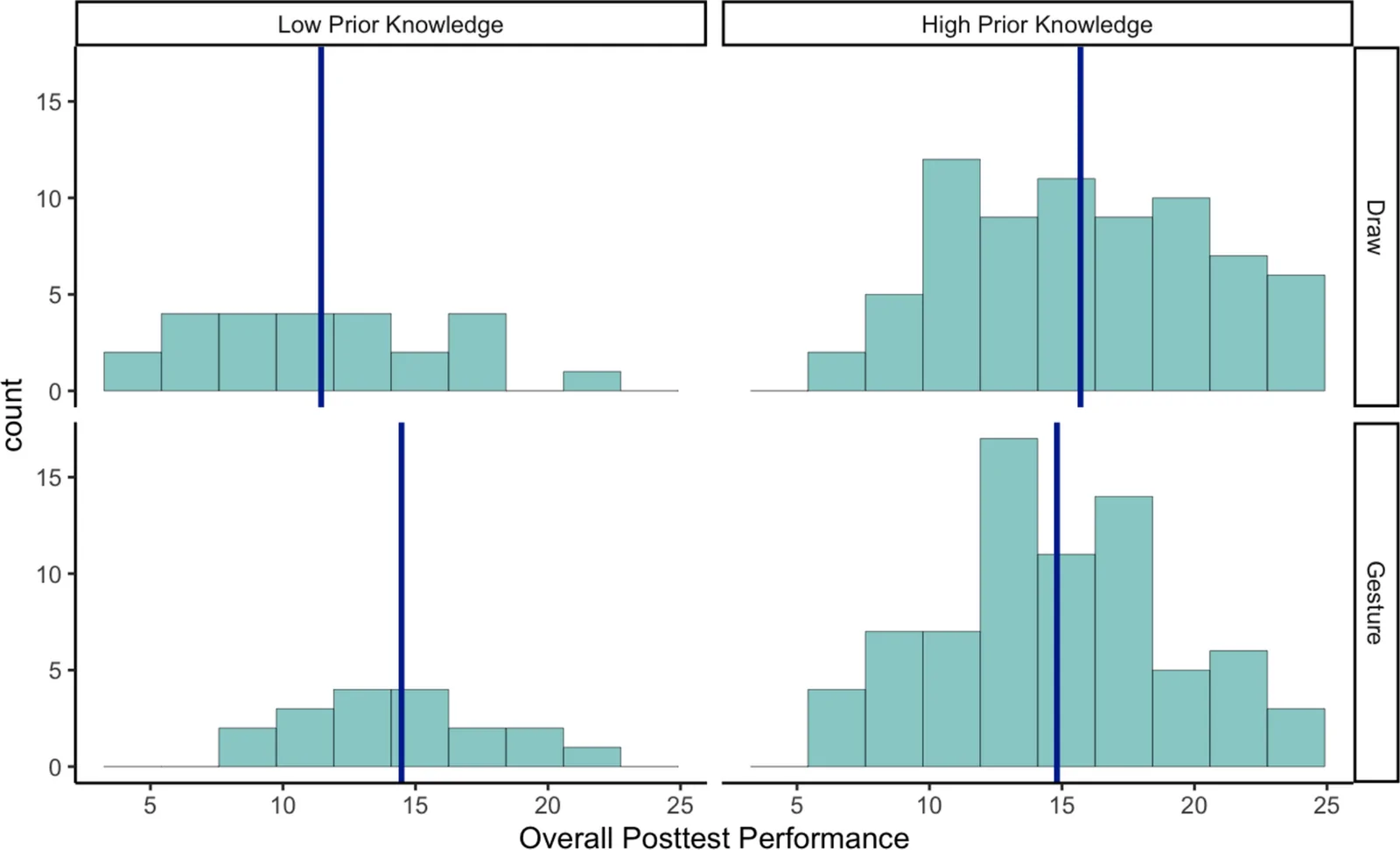
Low and high prior knowledge learners’ overall posttest performance by condition

We examined whether learners’ prior knowledge would moderate the effect of the type of embodied pedagogies on learners’ overall posttest performance (Fig. 2). A two-way ANOVA tested the effects of condition, prior knowledge, and their interaction on learners’ overall posttest performance (Table 1). The overall model was statistically significant, *F*(3, 184) = 5.46, *p* =.001, with a PRE of.08, indicating that approximately 8% of the variance in posttest scores was explained by the predictors. Importantly, there was a significant interaction between condition and prior knowledge, *F*(1, 184) = 6.04, *p* =.015, PRE =.03. This interaction suggests that the effectiveness of the instructional condition depended on learners’ prior knowledge.

**Table 1:** Results for the two-way interaction between condition and prior knowledge group using overall posttest performance as the outcome variable

Predictor	SS	*df*	MS	*F*	*p*	*PRE*
Model	337.90	3	112.63	5.46	.001	.08
Condition	96.22	1	96.22	4.66	.032	.02
Prior knowledge group	335.10	1	335.10	16.24	.000	.08
Condition*prior knowledge group	124.69	1	124.69	6.04	.015	.03
Error	3797.49	184	20.64			

**Fig. 3:**
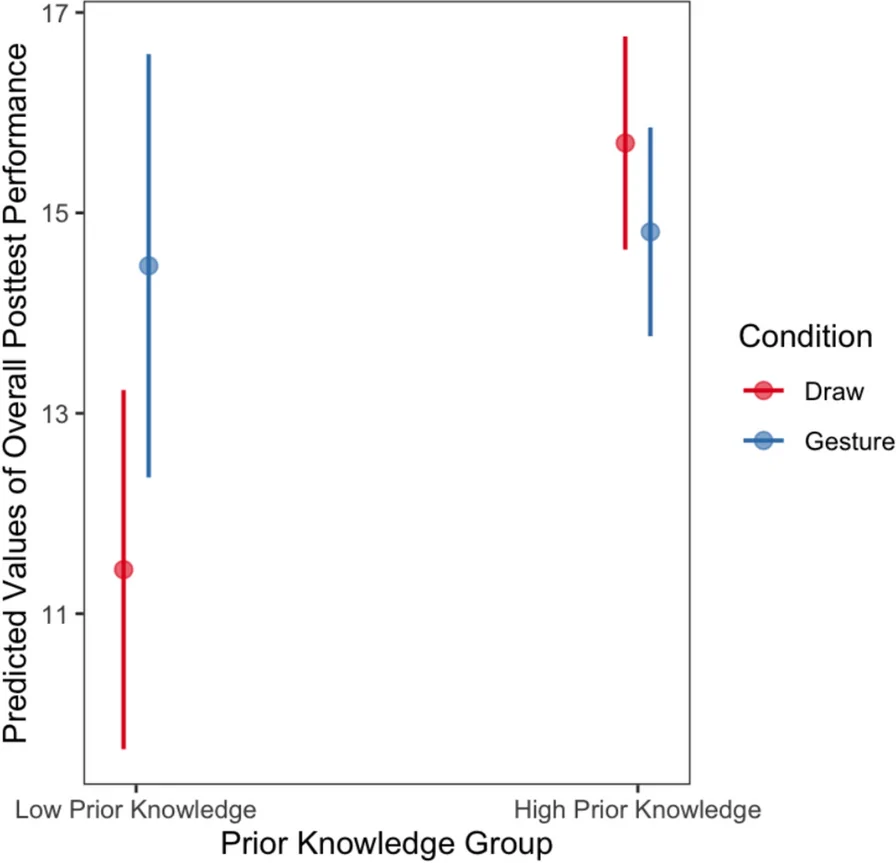
Marginal effects of the interaction between prior knowledge and condition

Inspection of the marginal means (Fig. 3) showed that consistent with our hypothesis, among learners with low prior knowledge, those in the Gesture group (M = 14.47, SD = 3.59) significantly outperformed learners in the Draw group (M = 11.44, SD = 4.74) on the overall posttest performance, *F*(1, 41) = 5.20, *p* =.028, PRE =.11. In contrast, for learners with high prior knowledge, there was no significant difference between learners in the Gesture group (M = 14.81, SD = 4.48) and Draw group (M = 15.70, SD = 4.74), *F*(1, 143) = 1.34,* p* =.249, PRE =.01.

### Exploratory analysis

In addition to the planned analyses, we further explored the effect of condition on students’ perceived engagement. A one-way ANOVA revealed that participants’ perceived engagement during the reenactment experience did not differ significantly between the two conditions, *F* (1,186) = 1.06, *p* =.305, PRE =.01.

**Fig. 4:**
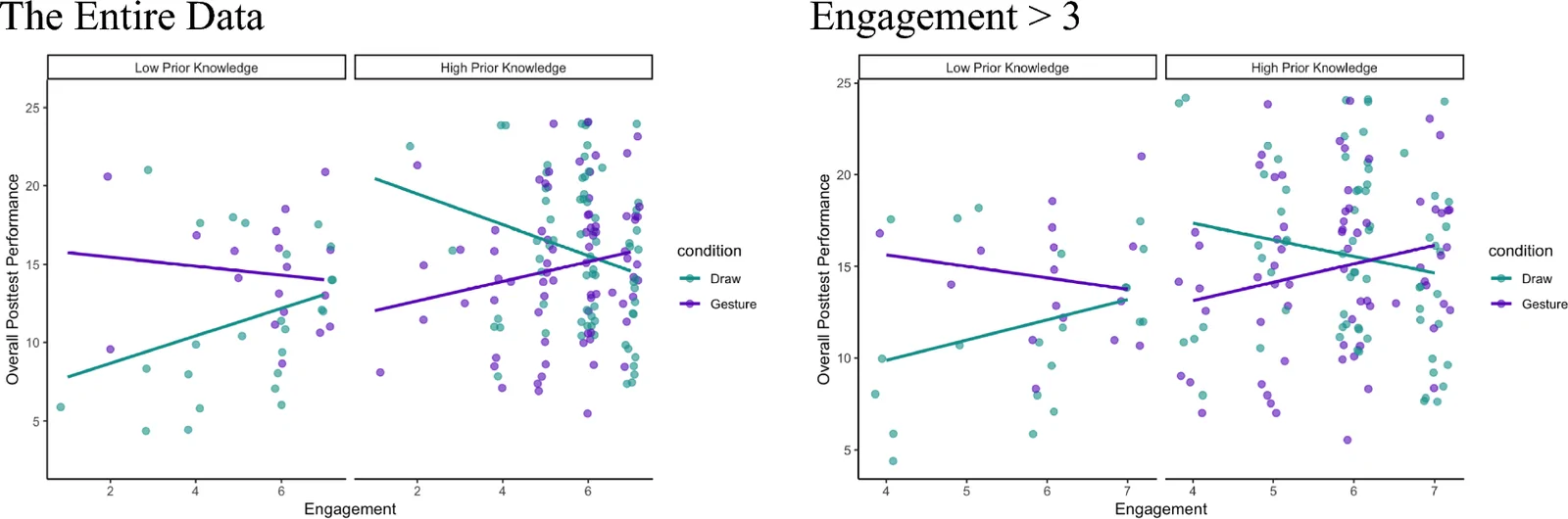
The relationship between engagement and overall posttest performance. *Note*. We included a graph for engagement >3 to avoid outliers biasing the pattern

Because engagement during reenactment may influence whether students benefit from embodied instructional activities, we next explored whether engagement further qualified the interaction between condition and prior knowledge in predicting posttest performance. As shown in Fig. 4, this pattern appeared to vary across levels of perceived engagement. We fit a linear model predicting posttest performance from condition, prior knowledge group, perceived engagement, and their three-way interactions. The complete model output is shown in Table 2. There was a significant three-way interaction between condition, prior knowledge, and engagement, which indicated that the two-way interaction between condition and prior knowledge might depend on learners’ perceived engagement during reenactment, *F*(1, 180) = 6.32, *p* =.013, PRE =.03.

**Table 2:** Results for the three-way interaction using overall posttest performance as the outcome variable

Predictor	SS	*df*	MS	*F*	*p*	PRE
(Intercept)	103.47	1	103.47	5.14	.025	–
Condition	64.24	1	64.24	3.19	.076	.02
Prior knowledge group	233.77	1	233.77	11.62	.001	.06
Engagement	49.04	1	49.04	2.44	.120	.01
Condition*Prior knowledge group	186.13	1	186.13	9.25	.003	.05
Condition*Engagement*	33.55	1	33.55	1.67	.198	.01
Prior knowledge group engagement	122.21	1	122.21	6.07	.015	.03
Condition*Prior knowledge Group*Engagement	127.18	1	127.18	6.32	.013	.03
Error	3622.23	180	20.12	–	–	–

To interpret the three-way interaction, estimated marginal means were computed at low (*M* − 1 *SD* = 4.25), average (*M* = 5.57), and high (*M* + 1 *SD* = 6.90) levels of perceived engagement using the emmeans package in R (Lenth, 2023). At low engagement, the interaction between condition and prior knowledge group was significant, *F*(1, 180) = 12.01, *p* <.001. Lower-prior-knowledge learners performed significantly better in the Gesture condition (*M* = 14.80, *SE* = 1.43) than in the Draw condition (*M* = 10.60, *SE* = 1.03), *t*(180) = 2.36, *p* =.019. In contrast, higher-prior-knowledge learners performed significantly better in the Draw condition (*M* = 17.30, *SE* = 0.97) than in the Gesture condition (*M* = 14.10, *SE* = 0.70), *t*(180) = 2.70, *p* =.008. At average engagement, the interaction remained significant, *F*(1, 180) = 5.35, *p* =.022, although neither within-prior-knowledge condition comparison reached significance (p >.05). At high engagement, the interaction was not significant, *F*(1, 180) < 0.01, *p* =.996. Because these follow-up comparisons were estimated from the same full linear model, all contrasts used the model residual degrees of freedom, *df* = 180.

## Discussion

The findings of this study contribute to an emerging literature examining how different forms of embodied pedagogies support the learning of abstract concepts in complex domains (Congdon et al., 2018; Congdon & Goldin-Meadow, 2021; Novack et al., 2014; Zhang et al., 2024b). Specifically, we compared two types of embodied pedagogies, gesturing and hand-drawing, that are commonly used by teachers in the classroom.

A central contribution of this study is the explicit consideration of learners’ prior knowledge as a moderating factor in the effectiveness of these pedagogies. Although prior knowledge has been recognized as important in embodied learning (e.g., Congdon & Goldin-Meadow, 2021; Congdon et al., 2018), this is the first study to directly compare gesture- and drawing-based instruction across learners with varying levels of prior knowledge. Results showed that learners with low prior knowledge benefited significantly more from gesture-based instruction than from drawing-based instruction. By contrast, no clear advantage emerged for drawing over gesture among high-prior-knowledge learners. The distributions of high-knowledge learners’ pre- and posttest scores suggest this null effect is unlikely due to a ceiling effect. Instead, it is possible that their domain knowledge enabled them to benefit from either form of embodied instruction, reducing the relative impact of the pedagogical differences.

Exploratory analyses that accounted for participants’ perceived engagement during the reenactment process suggested that the interaction between condition and prior knowledge group was most pronounced among relatively less engaged students and weakened as engagement increased. Specifically, among relatively less engaged students, those with lower prior knowledge showed higher posttest performance when they reenacted gestures than when they reenacted drawings, whereas those with higher prior knowledge showed higher posttest performance when they reenacted drawings than when they reenacted gestures. Among more engaged students, the effect of instructional condition appeared to depend less on students’ prior knowledge, and the two embodied instructional approaches resulted in more comparable posttest performance. However, because engagement was measured rather than experimentally manipulated, this exploratory finding should be interpreted descriptively rather than causally. In summary, we found that gesture-based instruction was especially beneficial for learners with lower prior knowledge, whereas exploratory analyses suggested that, among relatively less engaged students, lower-prior-knowledge learners performed better with gesture and higher-prior-knowledge learners performed better with drawing.

A potential explanation for the benefit of gesture-based instruction for low-prior-knowledge learners is that gesture might more tightly couple abstract concepts in bodily actions than drawing. Simulation-based theories of embodied cognition suggest that for embodied pedagogies to be effective, learners must construct multimodal representations that integrate sensory, motor, and conceptual information (e.g., Barsalou, 1999, 2008). For learners with low prior knowledge, gestures may more directly couple the structure of the concept to bodily movement.

In contrast, drawing introduces an additional representational layer: Learners must coordinate bodily action, a developing visual representation, and its intended conceptual interpretation. This added translation from bodily movement to an external artifact may not provide as effective sensorimotor grounding. Evidence consistent with this interpretation comes from Zacharia et al. (2012), who found that kindergarteners with limited prior knowledge learned more effectively from physically interacting with a balance scale than from manipulating a virtual simulation, where motor input was mediated through a computer interface. In both Zacharia et al. (2012) and our results, greater immediacy between action and conceptual consequence may be especially beneficial for novices.

The exploratory analysis may help us refine our interpretation. When learners are not highly engaged during reenactment, they may reproduce the gesture or drawing shown in the instructional video without actively reflecting on what the action represents. These conditions may make the mechanism described above especially consequential: When learners are not actively constructing meaning from the activity, learning may depend more heavily on the representational affordances of the embodied action itself. This is supported by findings showing that directed gestures can support learning without explicit awareness of their instructional purpose (Zhang et al., 2021). It is also notable that, among less engaged students with higher prior knowledge, drawing was associated with significantly higher posttest performance than gesture. Although exploratory, this finding suggests that drawing may be particularly useful when learners have enough prior knowledge to interpret and use the visual representation they produce, even when they are not highly engaged with the activity.

By contrast, higher engagement may have reduced learners’ dependence on the specific affordances of gesture versus drawing, such that the interaction between prior knowledge and the type of embodied pedagogies was no longer evident among more engaged students. Students who report higher engagement may be more likely to actively attend to and reflect on how the actions map onto the target concepts. As a result, both gesture and drawing may become viable routes for supporting learning, even for learners with lower prior knowledge.

### Situating the findings within prior work

At first glance, our findings might seem inconsistent with Congdon et al. (2018) who found that low-prior-knowledge children benefited more from object manipulation than from gesture-based instruction. However, these seemingly discrepant findings can be reconciled if we consider which of the two embodied pedagogies in the study more directly instantiates the concept being learned. In the Congdon et al. study, object manipulation may be more effective at directly instantiating the concepts in the bodily movements relative to gesture. For learners with very little prior knowledge, working with objects may therefore provide important support because the environment helps “hold” the conceptual structure for them, enabling potentially a tighter coupling between the actions and concepts. In our study, gesture might be more effective at directly instantiating the concepts with the movements relative to drawing. Unlike object manipulation, drawing may not provide the direct embodied experience that allows the experience to “hold” the structure but may provide a more indirect experience that requires additional meaning-making (Rau & Wu, 2018). In this sense, embodied pedagogies may lie along a continuum of representational directness in how performing (or even watching) bodily movements instantiate abstract concepts: Object manipulation may be more direct than gesture, which in turn is more direct than drawing in terms of representational distance from the concept.

Another possibility for bringing the findings together is to consider the developmental level of the learners. For younger learners, interaction with tangible objects that closely resemble everyday experiences may provide a strong grounding. In contrast, studies with more advanced learners have not consistently demonstrated advantages for object manipulation. For example, Beach et al. (2023) did not find benefits of object manipulation in terms of clay modeling over hand-drawing for medical students learning spinal anatomy.

### Limitations and future directions

The complex nature of the concepts targeted in this intervention presents both opportunities and challenges. Rather than examining the acquisition of a single concept, our study highlights how embodied pedagogies can support the learning of multiple, interrelated concepts. A key strength of the design is its ecological validity: Participants were concurrently enrolled in an introductory statistics and data science course, and the intervention resembled a mini-lecture aligned with their coursework. Unlike studies that rely on simplified stimuli with limited personal relevance, this intervention integrated directly with students’ actual learning context, increasing both engagement and potential for application. Importantly, the findings could be readily translated into instructional materials and classroom practices.

At the same time, recruiting students from real courses constrained the number of participants available. A natural next step would be to conduct larger-scale studies that more precisely isolate the mechanisms underlying our findings. Specifically, while our study compared gesture and drawing—two ecologically valid and widely used pedagogies—these approaches differ simultaneously in sensorimotor engagement and visuospatial permanence. Future research with a larger sample size could employ a two-way factorial design that independently manipulates sensorimotor engagement (high vs. low) and visuospatial permanence (high vs. low), allowing for a more precise test of their individual and combined effects.

Another challenge posed by the complexity of the concepts is the difficulty of separating learners by prior knowledge. Our approach—using an empirically derived threshold to distinguish between low- and high-prior-knowledge learners—provided a data-driven and context-specific criterion. Nonetheless, this data-driven approach is not always feasible and generalizable to other research and our measure of prior knowledge was limited to content-specific knowledge—that is, knowledge directly targeted by the intervention. Other forms of prior knowledge may also influence learners’ responsiveness to different pedagogies. These could include learners’ physical intuitions (e.g., Congdon et al., 2025), working memory (e.g., Congdon, 2024), and spatial reasoning abilities (e.g., Lee-Cultura & Giannakos, 2020). Future research should incorporate broader and more nuanced assessments of prior knowledge, ideally informed by philosophical and cognitive theories of grounded mental representations, to develop standardized and theory-driven methods for evaluating this construct.

Moreover, it is also important to note that in our study gesture and drawing were implemented in a structured watch–read–reenact sequence: Learners first observed the synchronized demonstration and narration, then read the sentence aloud, and finally reenacted the action. Speech–action synchrony may further strengthen learning by reinforcing links between linguistic input and sensorimotor simulation (Wagner et al., 2014), and it is therefore possible that tighter real-time alignment could amplify the effects observed here. At the same time, novices do not always know which movements are conceptually appropriate to generate independently. The watch–read–reenact structure provides a practical scaffold that guides learners’ actions while limiting working-memory demands. Because synchrony and movement presence were not manipulated in the present design, our findings should be interpreted as comparing gesture and drawing under this structured implementation. Future work could directly manipulate speech–action synchrony and include no-movement controls to examine whether these factors further shape the effectiveness of embodied instruction.

Relatedly, gesture and drawing differ in ways other than the coupling between the action and conceptual meaning and its translational cost. As we have emphasized in the introduction, this study cannot establish a causal interpretation of the mechanism. Rather, we started with two commonly used classroom pedagogies and hope to experimentally evaluate these embodied pedagogies for learners with different levels of prior knowledge. These insights serve as a working hypothesis and a stepping stone for future research to experimentally isolate different mechanisms at work.

Lastly, to further enhance both the ecological validity and generalizability of our findings, we encourage future studies to examine the effectiveness of embodied pedagogies in authentic, longitudinal classroom environments. Our study took a first step by recruiting students enrolled in an introductory statistics and data science course, who had a relatively high intrinsic motivation to learn. Building on this, future work might be interested in extending the paradigm to an authentic classroom setting across different age-groups and test the effectiveness of such pedagogies over a longer period of time. A longitudinal design, in particular, would offer a valuable opportunity to track the natural progression of learners’ conceptual understanding and explore how different features of embodied instruction contribute at various stages of knowledge development. For instance, certain pedagogical features may be especially beneficial during the initial stages of learning, while others may become more impactful as learners’ understanding deepens. Such insights could inform adaptive use of embodied strategies across instructional timelines to better support diverse learners’ needs.

In summary, this study is the first to compare the effectiveness of gesture and hand-drawing for teaching modeling-related concepts to learners with varying levels of prior knowledge. Our findings, particularly the observed two-way interaction between learners’ prior knowledge and the type of embodied pedagogies, highlight the importance of considering learners’ prior knowledge when designing and implementing embodied pedagogies.

## Data Availability

The study design, hypotheses, and analytic plans were not preregistered. The study data can be accessed through: https://osf.io/zth3g/?view_only=4539759962164c8085b3acda1d854ad4, and the analysis code can be provided upon request.
